# Association of obesity-related blood chemistry alterations with animal welfare outcomes in sterilized cats: A case-control study in Indonesia

**DOI:** 10.14202/vetworld.2026.2531-2544

**Published:** 2026-06-20

**Authors:** Sugeng Dwi Hastono, Widagdo Sri Nugroho, Guntari Titik Mulyani

**Affiliations:** 1 Student of Magister Veterinary Science Study Program, Faculty of Veterinary Medicine, Universitas Gadjah Mada, Yogyakarta, Indonesia; 2 Department of Veterinary Public Health, Faculty of Veterinary Medicine, Universitas Gadjah Mada, Yogyakarta, Indonesia; 3 Department of Internal Medicine, Faculty of Veterinary Medicine, Universitas Gadjah Mada, Yogyakarta, Indonesia

**Keywords:** animal welfare, blood chemistry, body condition score, feline obesity, housing management, Indonesia, sterilized cats, triglycerides

## Abstract

**Background and Aim:**

Obesity is a common health concern in sterilized cats and may adversely affect animal welfare through metabolic disturbances and increased susceptibility to disease. Although sterilization offers several health and population-control benefits, it can predispose cats to excessive weight gain. This study investigated the association between obesity-related alterations in blood chemistry and animal welfare outcomes in sterilized cats by comparing obese and ideal body condition score (BCS) groups in Lampung, Indonesia.

**Materials and Methods:**

An unmatched case-control study was conducted from May to July 2025 at a veterinary clinic in Lampung Province, Indonesia. A total of 104 sterilized cats were enrolled, comprising 52 overweight-obese cats (BCS 6–9) and 52 cats with ideal body condition (BCS 4–5). Data were collected through owner questionnaires, clinical examinations, welfare assessments, and blood sampling. Blood glucose, aspartate aminotransferase (AST), alanine aminotransferase (ALT), triglycerides, and creatinine concentrations were analyzed. Associations between obesity status, welfare indicators, risk factors, and blood chemistry profiles were evaluated using Chi-square and logistic regression analyses.

**Results:**

Most cat owners were female, lived in urban areas, and possessed undergraduate-level education. Male cats predominated in the study it was significantly associated with overweight-obesity (p = 0.049). Deworming status (p = 0.042), anti-ectoparasite treatment (p = 0.024), housing system (p = 0.006), and cage management (p = 0.045) were also significantly associated with obesity status. Clinical evaluations revealed generally favorable welfare outcomes, with low prevalence of integumentary, digestive, respiratory, and urogenital disorders. Ectoparasite infestation was significantly associated with obesity status (p = 0.011). Blood glucose, AST, ALT, triglyceride, and creatinine concentrations did not differ significantly between obese and ideal-BCS cats (p > 0.05). Although elevated triglyceride and ALT values were observed in some animals, these abnormalities occurred in both groups without significant differences.

**Conclusion:**

Sterilized obese cats exhibited blood chemistry profiles comparable to those of cats with ideal BCS, suggesting physiological adaptation and maintenance of metabolic homeostasis. Nevertheless, obesity was associated with management-related factors, including housing practices and parasite control. Overall, favorable owner awareness and husbandry practices contributed to satisfactory animal welfare outcomes, although improved attention to dental health and preventive care remains necessary.

## INTRODUCTION

Animal welfare, according to the World Organisation for Animal Health (WOAH), refers to the physical and mental state of an animal in relation to the conditions in which it lives and dies [1]. In companion animals, animal welfare is closely associated with the concept of the Five Freedoms. The World Small Animal Veterinary Association (WSAVA) emphasizes that companion animals should be allowed to express normal behaviors, be housed appropriately with or apart from other animals as required, and be protected from pain, suffering, injury, and disease through adequate health care and management practices [2].

The domestic cat (*Felis catus*) is among the most popular companion animals worldwide. The number of households keeping cats increased substantially during the coronavirus disease 2019 (COVID-19) pandemic, with reports indicating increases of up to 250%, largely due to their positive effects in reducing loneliness and improving mental well-being [3]. This growing population has increased owners’ responsibility to understand and implement appropriate animal welfare practices [4]. Many owners rely on online resources for information regarding cat care; however, the accuracy and reliability of such information are often variable, particularly regarding animal welfare and health management [5]. One of the most commonly discussed topics is sterilization, or gonadectomy, including castration in male cats and ovariohysterectomy in female cats. Sterilization is widely recommended because it reduces unwanted reproduction, decreases aggressive and territorial behaviors, lowers the risk of reproductive tract diseases, and contributes to cancer prevention [6].

Despite its numerous benefits, sterilization is associated with physiological and metabolic changes that may predispose cats to excessive weight gain. Basal metabolic rate decreases following sterilization, resulting in reduced physical activity and altered appetite regulation. Increased food intake commonly peaks approximately 8–10 weeks after surgery [7, 8]. Sterilized cats exhibit reduced estradiol production, an important regulator of energy intake, leading to hyperphagia and increased body weight [9, 10]. In addition, reductions in leptin activity and lipoprotein lipase function contribute to increased appetite and altered lipid metabolism, resulting in elevated circulating fatty acid concentrations [11, 12]. Because energy requirements decrease after sterilization, failure to adjust dietary management appropriately may lead to increased body condition and the development of overweight or obesity [9].

The WSAVA body condition scoring system identifies a body condition score (BCS) of 5 on a 9-point scale as ideal, whereas scores of 6–9 indicate overweight to obese conditions [13]. The BCS assessment is a practical and widely accepted method for evaluating body fat accumulation and overall health status in cats [14]. Gonadectomy has also been associated with increased interleukin-6 production and activation of protein kinase pathways that influence insulin receptor function, potentially contributing to insulin resistance and elevated blood glucose concentrations [11, 12]. Increased BCS has been linked to numerous adverse health outcomes, including atopic dermatitis, arthritis, hypertension, asthma, diarrhea, lower urinary tract disorders, ocular diseases, diabetes mellitus, allergies, and reduced life expectancy [15]. Furthermore, overweight and obesity are associated with an increased risk of mortality and the development of metabolic disorders such as diabetes mellitus and hepatic lipidosis, as well as chronic conditions that impair quality of life, including osteoarthritis [16].

Larsen [17] reported that reductions in sex hormone concentrations following sterilization disrupt lipid metabolism, reducing the body’s ability to convert triglycerides into fatty acids and glycerol and promoting the accumulation of white adipose tissue in multiple organs. Progressive weight gain may contribute to increased vascular resistance and hypertension [17]. Persistent hypertension can induce structural and functional alterations in blood vessels, endothelial dysfunction, and oxidative stress, leading to vasoconstriction of the efferent arterioles, glomerular hypertension, proteinuria, and eventual renal impairment. These pathological processes may be reflected by increased serum creatinine concentrations [18]. Consequently, obesity in sterilized cats may represent an important health concern with direct implications for animal welfare.

Although the physiological consequences of obesity and the benefits of sterilization have been extensively investigated, limited information is available regarding the relationship between obesity-associated metabolic alterations and overall animal welfare outcomes in sterilized cats under routine household management conditions. Previous studies have primarily focused on obesity prevalence, risk factors, or individual disease conditions, whereas the combined assessment of welfare indicators, management practices, and blood chemistry profiles in sterilized cats remains insufficiently explored. Moreover, evidence from Indonesia on the interactions among obesity, metabolic health, and welfare outcomes in companion cats is scarce. Understanding these relationships is essential for developing evidence-based recommendations that promote both health and welfare in sterilized companion animals.

Therefore, this study aimed to compare blood chemistry profiles and animal welfare indicators between obese and ideal-BCS sterilized cats in Lampung, Indonesia. Specifically, the study evaluated the associations between obesity status, management-related factors, clinical welfare indicators, and selected blood chemistry parameters, including blood glucose, aspartate aminotransferase (AST), alanine aminotransferase (ALT), triglycerides, and creatinine. The findings are expected to provide valuable information regarding the welfare implications of obesity in sterilized cats and support the development of effective preventive and management strategies for companion animal health and welfare.

## MATERIALS AND METHODS

### Ethical approval

This study was reviewed and approved by the Research Ethics Commission, Faculty of Veterinary Medicine, Universitas Gadjah Mada, Yogyakarta, Indonesia (Approval No. 50/EC-FKH/int./2025; April 28, 2025). The study was conducted in accordance with institutional ethical guidelines governing research involving animal owners and companion animals. All participating cat owners received detailed information about the study’s objectives, procedures, benefits, and potential risks before enrollment. Written informed consent was obtained from all participants before questionnaire administration, clinical examination of their cats, and blood sample collection.

### Study period and location

The study was conducted from May to July 2025 at Amanah Veterinary Services, a companion-animal veterinary clinic in Lampung Province, Indonesia. Participant recruitment, clinical examinations, questionnaire administration, welfare assessments, and blood sample collection were conducted throughout the study period.

### Study design

**Figure 1:**
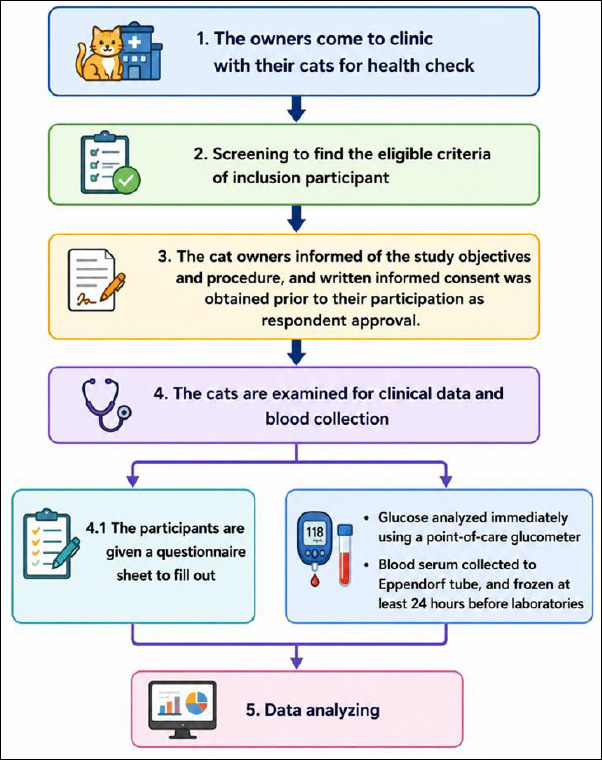
Flow chart of the study.

An unmatched case-control study was conducted to evaluate the association between obesity-related alterations in blood chemistry and animal welfare outcomes in sterilized cats. Consecutive sampling was used to recruit eligible participants and minimize selection bias. Data were collected at a single point in time and included questionnaire responses, clinical examination findings, welfare assessments, and blood chemistry measurements. The overall study workflow is presented in Figure 1.

### Participants

Eligible cats were sterilized >3 months before enrollment (verified through owner records), aged ≥6 months, and had a BCS assessed using the WSAVA 9-point body condition scoring system [7, 8]. Cats with a BCS of 6–9 were classified as cases (overweight-obese), whereas cats with a BCS of 4–5 were classified as controls (ideal body condition).

Cats were excluded if they had known chronic diseases, including diabetes mellitus, hyperthyroidism, chronic kidney disease, or hepatic disease; a history of illness within the previous 4 weeks; current administration of glucocorticoids or other medications affecting metabolism; or incomplete medical records. Potential sources of selection, recall, and information bias were minimized through consecutive recruitment and standardized data collection procedures.

Cases and controls were selected consecutively from clinic records and client visits until the required sample size was achieved. No matching was performed between groups.

Sample size was calculated using Epitools (https://epitools.ausvet.com.au/casecontrolss). Assuming a 3% prevalence of the primary exposure among controls, an odds ratio (OR) of 10, a 95% confidence level, and 90% power, the minimum required sample size was estimated at 52 cats per group. This sample size was considered adequate to detect moderate-to-large associations between obesity status and the investigated risk factors.

### Data and sample collection

Before the commencement of the study, the questionnaire was pilot-tested with 10 cat owners to assess its clarity and comprehensibility. Minor modifications were made to improve wording and consistency.

Owners of eligible cats completed a structured questionnaire. Information collected included owner demographics (age, sex, educational background, place of residence, and cat ownership experience), cat characteristics (breed, sex, age, vaccination status, deworming history, and anti-ectoparasite treatment history), management practices (diet type, feeding regimen, housing system, and duration of owner-cat interaction), and welfare-related practices.

All clinical examinations were performed by a single licensed veterinarian with training in animal welfare assessment. BCS was assessed using the WSAVA 9-point BCS chart [13]. Dental and periodontal status were evaluated using a simplified 0–3 scoring system, where 0 indicated normal findings and 3 indicated severe disease. Ectoparasite infestation was assessed through visual inspection and combing, whereas endoparasite infection was evaluated by direct fecal examination under a microscope.

### Blood sample collection and laboratory analysis

Blood samples (approximately 2–3 mL) were collected from the cephalic vein using a 23–25 G needle and a 3 mL syringe under minimal restraint to reduce stress. Cats were not routinely fasted before sampling; however, fasting status was recorded when available.

Blood glucose concentrations were measured immediately after collection using a point-of-care glucometer (Autocheck 3 in 1; General Life Biotechnology Co., Ltd., Taipei, Taiwan; serial number BD04014997). Point-of-care glucose measurements may differ slightly from laboratory values because of device-specific calibration characteristics.

Whole blood samples were allowed to clot at room temperature for 1–2 h to obtain serum. The serum was then transferred aseptically into sterile Eppendorf tubes and stored frozen for at least 24 h before laboratory analysis.

Serum biochemical analyses, including AST, ALT, triglycerides, and creatinine concentrations, were performed using a MICROLAB 300 semi-automated analyzer (ELITech Group, Puteaux, France) according to the manufacturer’s instructions at the Disease Investigation Center (Balai Veteriner Lampung), Bandar Lampung, Lampung Province, Indonesia. Daily quality-control procedures were conducted using commercial control materials.

Reference intervals established by the Balai Veteriner Lampung Laboratory for adult cats were used for interpretation of results: blood glucose, 70–150 mg/dL; AST, 8.9–48.5 U/L; ALT, 8.2–57.3 U/L; triglycerides, 10–150 mg/dL; and creatinine, 0.6–2.4 mg/dL. Values were categorized as low, normal, or high according to these reference intervals.

### Statistical analysis

Data were entered into Microsoft Excel (Microsoft Corporation, Redmond, WA, USA) and analyzed using IBM SPSS Statistics version 24 (IBM Corporation, Armonk, NY, USA). Descriptive statistics are presented as frequencies and percentages for categorical variables and as mean ± SD or median (IQR) for continuous variables, depending on data distribution. Normality was assessed using the Shapiro-Wilk test.

Associations between obesity status and categorical risk factors or blood chemistry categories were evaluated using Pearson’s Chi-square test or Fisher’s exact test, as appropriate. The strength of association was expressed as crude ORs with corresponding 95% CI.

Multivariable logistic regression analysis was performed to control for potential confounding variables, including age, sex, and diet type. Statistical significance was established at p < 0.05. No adjustment for multiple comparisons was applied because of the exploratory nature of the study. Missing data were handled using complete-case analysis.

## RESULTS

### Demographic characteristics of participants

The demographic characteristics of the participating cat owners are summarized in Table 1. Most participants were from Bandar Lampung City, an urban area of Lampung Province (68.3%; 36.5% cases and 31.7% controls). Most owners were female (81.7%; 42.3% cases and 39.4% controls) and had an undergraduate-level educational background (76.9%; 39.4% cases and 37.5% controls). Approximately half of the participants belonged to the millennial generation, aged 24–39 years (43.3%; 19.3% cases and 24.0% controls). Most owners had ≥5 years of experience keeping cats (80.8%; 37.5% cases and 43.3% controls), kept ≥2 cats (94.2%; 47.1% cases and 47.1% controls), and kept both male and female cats (85.5%; 41.3% cases and 44.2% controls). Vaccination awareness was high, with 95.2% of owners reporting that their cats were vaccinated, while only 4.8% reported that their cats were not.

**Table 1:** Demographic characteristics of cat owners in the case-control study in Lampung (N = 104)

Variable	Case, n (%)	Control, n (%)	Total, n (%)
Origin			
Bandar Lampung (urban)	38 (36.5)	33 (31.7)	71 (68.3)
Lampung Selatan (rural)	8 (7.7)	16 (15.3)	24 (23.1)
Lampung Tengah (rural)	1 (0.95)	1 (0.95)	2 (1.9)
Lampung Timur (rural)	0 (0.0)	1 (1.0)	1 (1.0)
Pesawaran (rural)	3 (2.9)	1 (1.0)	4 (3.8)
Pringsewu (rural)	2 (1.9)	0 (0.0)	2 (1.9)
Gender			
Male	8 (7.7)	11 (10.6)	19 (18.3)
Female	44 (42.3)	41 (39.4)	85 (81.7)
Education level			
Senior high school	11 (10.6)	13 (12.5)	24 (23.1)
Undergraduate	41 (39.4)	39 (37.5)	80 (76.9)
Generation (age)			
Gen Z (8–23 years)	8 (7.7)	10 (9.6)	18 (17.3)
Millennial (24–39 years)	20 (19.3)	25 (24.0)	45 (43.3)
Gen X (40–55 years)	14 (13.4)	12 (11.6)	26 (25.0)
Baby boomer (56–76 years)	10 (9.6)	5 (4.8)	15 (14.4)
Experience keeping cats			
<5 years	13 (12.5)	7 (6.7)	20 (19.2)
≥5 years	39 (37.5)	45 (43.3)	84 (80.8)
Number of cats			
1 cat	3 (2.9)	3 (2.9)	6 (5.8)
≥2 cats	49 (47.1)	49 (47.1)	98 (94.2)
Sex of cats			
Male only or female only	9 (8.7)	6 (5.8)	15 (14.5)
Both male and female	43 (41.3)	46 (44.2)	89 (85.5)
Vaccination awareness			
No	1 (1.0)	4 (3.8)	5 (4.8)
Yes	51 (49.0)	48 (46.2)	99 (95.2)

### Cat-related risk factors associated with obesity status

Cat-related risk factors associated with obesity status are presented in Table 2. The most common breed was the domestic cat (40.4%; 23.1% cases and 17.3% controls). Male cats predominated in the study population (72.1%; 40.4% cases and 31.7% controls). Cat sex was significantly associated with obesity status (χ² = 3.873, p = 0.049), with male cats showing a higher proportion of cases than female cats. The OR for obesity in male cats was 2.418, with a 95% CI of 0.992–5.896.

**Table 2:** Cat-related risk factors associated with obesity status in the case-control study in Lampung (N = 104)

Criteria	Case, n (%)	Control, n (%)	Total, n (%)	χ² (p-value)	OR	95% Confidence interval
Cat breed				8.211 (0.314)	–	–
Persia	14 (13.4)	12 (11.5)	26 (25.0)			
Domestic	24 (23.1)	18 (17.3)	42 (40.4)			
Himalayan	0 (0.0)	1 (1.0)	1 (1.0)			
Maine Coon	0 (0.0)	2 (1.9)	2 (1.9)			
Bengal	0 (0.0)	1 (1.0)	1 (1.0)			
Ragdoll	1 (1.0)	0 (0.0)	1 (1.0)			
Balinese	1 (1.0)	0 (0.0)	1 (1.0)			
Domestic cross	12 (11.5)	18 (17.3)	30 (28.8)			
Cat sex				3.873 (0.049)*	2.418	0.992–5.896
Male	42 (40.4)	33 (31.7)	75 (72.1)			
Female	10 (9.6)	19 (18.3)	29 (27.9)			
Vaccination status				3.651 (0.161)	–	–
Unvaccinated	8 (7.7)	16 (15.4)	24 (23.1)			
Vaccinated	44 (42.3)	36 (34.6)	80 (76.9)			
Deworming status				6.362 (0.042)*	0.337	0.084–1.349
Absent	3 (2.9)	8 (7.7)	11 (10.6)			
Yes	49 (47.1)	44 (42.3)	93 (89.4)			
Anti-ectoparasite status				7.446 (0.024)*	0.311	0.092–1.050
Absent	4 (3.8)	11 (10.6)	15 (14.4)			
Yes	48 (46.1)	41 (39.4)	89 (85.6)			

* p < 0.05.

Of the 104 cats, 24 (23.1%) were unvaccinated and 80 (76.9%) were vaccinated. Vaccination status was not significantly associated with obesity status (χ² = 3.651, p = 0.161). Most cats had received deworming treatment (89.4%; 47.1% cases and 42.3% controls), and deworming status was significantly associated with obesity status (χ² = 6.362, p = 0.042; OR = 0.337; 95% CI = 0.084–1.349). Similarly, most cats had received anti-ectoparasite treatment (85.6%; 46.1% cases and 39.4% controls), and anti-ectoparasite treatment status was significantly associated with obesity status (χ² = 7.446, p = 0.024; OR = 0.311; 95% CI = 0.092–1.050).

Management-related risk factors associated with obesity status are presented in Table 3. All cats received dry food. Among them, 46 cats received dry food supplemented with wet food (44.3%; 23.1% cases and 21.2% controls), and 72 cats had food available at all times (69.3%; 36.6% cases and 32.7% controls). All cats had continuous access to drinking water, and most cats used a drinking bowl (91.4%; 45.2% cases and 46.2% controls).

**Table 3:** Management-related risk factors associated with obesity status in the case-control study in Lampung (N = 104)

Criteria	Case, n (%)	Control, n (%)	Total, n (%)	χ² (p-value)	OR	95% Confidence interval
Type of feed				1.173 (0.760)	–	–
Dry food	8 (7.7)	12 (11.5)	20 (19.2)			
Dry food + wet food	24 (23.1)	22 (21.2)	46 (44.3)			
Dry food + homemade food	8 (7.7)	6 (5.8)	14 (13.5)			
Dry food + wet food + homemade food	12 (11.5)	12 (11.5)	24 (23.0)			
Feeding frequency				3.374 (0.185)	–	–
2–3 times/day	7 (6.7)	14 (13.5)	21 (20.2)			
4–5 times/day	7 (6.7)	4 (3.8)	11 (10.5)			
Always available	38 (36.6)	34 (32.7)	72 (69.3)			
Drinking water system				0.122 (0.727)	–	–
Drinking bowl	47 (45.2)	48 (46.2)	95 (91.4)			
Water fountain	5 (4.8)	4 (3.8)	9 (8.6)			
Housing system				7.505 (0.006)*	2.156	1.740–2.670
Kept free outdoors	0 (0.0)	7 (6.7)	7 (6.7)			
Kept free indoors	52 (50.0)	45 (43.3)	97 (93.3)			
Cage system				3.983 (0.045)*	0.220	0.044–1.091
Uncaged	44 (42.3)	50 (48.1)	94 (90.4)			
Caged	8 (7.7)	2 (1.9)	10 (9.6)			
Cat nature				4.397 (0.222)	–	–
Lazy	7 (6.7)	7 (6.7)	14 (13.4)			
Active	45 (43.3)	45 (43.3)	90 (86.6)			
Owner interaction				0.796 (0.672)	–	–
No petting/activity	7 (6.7)	10 (9.6)	17 (16.3)			
Petting and play	45 (43.3)	42 (40.4)	87 (83.7)			
Daily activity duration				2.648 (0.266)	–	–
<15 min/day	14 (13.5)	17 (16.3)	31 (29.8)			
≥15 min/day	38 (36.5)	35 (33.7)	73 (70.2)			

* p < 0.05.

Most cats were described as active (86.6%; 43.3% cases and 43.3% controls), whereas 13.4% were described as lazy. Most cats received petting and play from their owners (83.7%; 43.3% cases and 40.4% controls), and 70.2% received >15 min of daily owner interaction. The housing system was significantly associated with obesity status (χ² = 7.505, p = 0.006; OR = 2.156; 95% CI = 1.740–2.670), with most cats kept freely indoors. Cage system was also significantly associated with obesity status (χ² = 3.983, p = 0.045; OR = 0.220; 95% CI = 0.044–1.091), with most cats maintained without cages.

According to the WSAVA animal welfare guidelines, the basic welfare requirements of companion animals include a suitable environment, an appropriate diet, the ability to express normal behavior, appropriate housing, whether with or apart from other animals, and protection from pain, suffering, injury, and disease [2]. In this study, health status was defined as the absence of clinically detectable disease affecting major organ systems or normal physiological function due to pathogens, fungi, parasites, wounds, or trauma.

### Clinical findings

The clinical examination findings are presented in Table 4. Most cats had no integumentary disease (88.5%; 42.3% cases and 46.2% controls), digestive disease (94.3%; 46.2% cases and 48.1% controls), urogenital disease (96.2%; 48.1% cases and 48.1% controls), or respiratory disease (90.3%; 43.3% cases and 47.0% controls). Dental and periodontal disorders were recorded in 70 cats (67.3%; 34.6% cases and 32.7% controls). Almost all cats had no endoparasite infection (99.0%; 49.0% cases and 50.0% controls). Ectoparasites were detected in 55 cats (52.9%; 20.2% cases and 32.7% controls), and ectoparasite status was significantly associated with obesity status (χ² = 6.522, p = 0.011; OR = 2.788; 95% CI = 1.258–6.179).

**Table 4:** Clinical findings of cats in the case-control study in Lampung (N = 104).

Parameter	Case, n (%)	Control, n (%)	Total, n (%)	χ² (p-value)	OR	95% Confidence interval
Integumentary disease				1.507 (0.220)	–	–
Absent	44 (42.3)	48 (46.2)	92 (88.5)			
Present	8 (7.7)	4 (3.8)	12 (11.5)			
Digestive disease				0.707 (0.400)	–	–
Absent	48 (46.2)	50 (48.1)	98 (94.3)			
Present	4 (3.8)	2 (1.9)	6 (5.7)			
Urogenital disease				0.000 (1.000)	–	–
Absent	50 (48.1)	50 (48.1)	100 (96.2)			
Present	2 (1.9)	2 (1.9)	4 (3.8)			
Respiratory disease				1.770 (0.183)	–	–
Absent	45 (43.3)	49 (47.0)	94 (90.3)			
Present	7 (6.7)	3 (3.0)	10 (9.7)			
Dental and periodontal disease				2.904 (0.574)	–	–
Absent	16 (15.4)	18 (17.3)	34 (32.7)			
Present	36 (34.6)	34 (32.7)	70 (67.3)			
Ectoparasites				6.522 (0.011)*	2.788	1.258–6.179
Absent	31 (29.8)	18 (17.3)	49 (47.1)			
Present	21 (20.2)	34 (32.7)	55 (52.9)			
Endoparasites				1.010 (0.315)	–	–
Absent	51 (49.0)	52 (50.0)	103 (99.0)			
Present	1 (1.0)	0 (0.0)	1 (1.0)			

* p < 0.05.

### Blood chemistry profile

The blood chemistry profiles are presented in Table 5. Blood glucose concentrations were within the normal range in 84 cats (79.0%; 40.4% cases and 38.6% controls). In the case group, 4 cats (3.8%) had low glucose values and 4 cats (3.8%) had high glucose values. In the control group, 6 cats (5.7%) had low glucose values and 6 cats (5.7%) had high glucose values. Blood glucose category did not differ significantly between cases and controls (χ² = 0.990, p = 0.609).

**Table 5:** Blood chemistry profile of cats in the case-control study in Lampung (N = 104).

Parameter	Case, n (%)	Control, n (%)	Total, n (%)	χ² (p-value)
Blood glucose				0.990 (0.609)
Low	4 (3.8)	6 (5.7)	10 (9.5)	
Normal	44 (40.4)	40 (38.6)	84 (79.0)	
High	4 (3.8)	6 (5.7)	10 (9.5)	
AST				1.870 (0.393)
Low	0 (0.0)	1 (1.0)	1 (1.0)	
Normal	47 (45.2)	43 (41.3)	90 (86.5)	
High	5 (4.8)	8 (7.7)	13 (12.5)	
ALT				1.419 (0.492)
Low	6 (5.8)	7 (6.8)	13 (12.6)	
Normal	30 (28.8)	24 (23.0)	54 (51.8)	
High	16 (15.4)	21 (20.2)	37 (35.6)	
Triglycerides				1.024 (0.599)
Low	10 (9.6)	9 (8.6)	19 (18.2)	
Normal	18 (17.3)	14 (13.5)	32 (30.8)	
High	24 (23.1)	29 (27.9)	53 (51.0)	
Creatinine				3.829 (0.050)
Low	46 (44.2)	51 (49.0)	97 (93.2)	
Normal	6 (5.8)	1 (1.0)	7 (6.8)	

AST = Aspartate aminotransferase, ALT = Alanine aminotransferase. * p < 0.05.

AST concentrations were within the normal range in 90 cats (86.5%; 45.2% cases and 41.3% controls). In the case group, 5 cats (4.8%) had high AST values. In the control group, 1 cat (1.0%) had a low AST value and 8 cats (7.7%) had high AST values. AST category did not differ significantly between cases and controls (χ² = 1.870, p = 0.393).

ALT concentrations were within the normal range in 54 cats (51.8%; 28.8% cases and 23.0% controls). In the case group, 6 cats (5.8%) had low ALT values and 16 cats (15.4%) had high ALT values. In the control group, 7 cats (6.8%) had low ALT values and 21 cats (20.2%) had high ALT values. ALT category did not differ significantly between cases and controls (χ² = 1.419, p = 0.492).

Triglyceride concentrations were within the normal range in 32 cats (30.8%; 17.3% cases and 13.5% controls). In the case group, 10 cats (9.6%) had low triglyceride levels and 24 (23.1%) had high triglyceride levels. In the control group, 9 cats (8.6%) had low triglyceride values and 29 (27.9%) had high triglyceride values. Triglyceride category did not differ significantly between cases and controls (χ² = 1.024, p = 0.599).

Creatinine concentrations were within the normal range in 7 cats (6.8%; 5.8% cases and 1.0% controls). Low creatinine values were observed in 46 cats (44.2%) in the case group and 51 cats (49.0%) in the control group. Creatinine category was not significantly different between cases and controls, although the p-value was at the threshold of significance (χ² = 3.829, p = 0.050).

## DISCUSSION

### Demographics of cat owners

Cat ownership in Lampung was predominantly reported among women (81.7%), particularly those residing in Bandar Lampung City (68.3%), followed by Lampung Selatan (23.1%), a rural area near Bandar Lampung. Most participants had an undergraduate educational background (76.9%), whereas 23.1% had completed senior high school. Approximately half of the participants (43.3%) belonged to the millennial generation (24–39 years), followed by Generation X (25.0%; 40–55 years). Consistent with their age profile, most participants had extensive experience in cat ownership, with 80.8% having kept cats for >5 years. In addition, most owners kept multiple cats, with 94.2% owning ≥2 cats and 85.5% keeping both male and female cats.

Paul *et al*. [19] similarly reported that most female pet owners owned cats (79.03%), approximately half of the owners had undergraduate-level education (51.61%), and most belonged to the 18–30-year age group (56.45%). Furthermore, 66.13% of cat owners had previous experience in pet rearing [19]. The demographic characteristics observed in the present study suggest that educational background, urban residence, and experience in pet ownership may positively influence awareness and implementation of animal welfare practices.

### Risk factors associated with obesity in sterilized cats

As shown in Table 2, cat breed, vaccination status, type of feed, feeding frequency, drinking water system, cat temperament, owner interaction, and daily activity duration were not significantly associated with overweight or obesity status. Similar findings were reported by Paul *et al*. [19], who found that most cats were local breeds (71.43%), most owners fed home-prepared diets (72.58%), and female cats predominated in the study population (88.57%).

In contrast, cat sex was significantly associated with overweight-obesity status (p = 0.049), with male cats showing a greater likelihood of being overweight or obese. Male cats have previously been reported to possess a higher predisposition to excessive weight gain than females [20]. Following sterilization, reduced estradiol production alters energy intake regulation and promotes hyperphagia, resulting in increased body weight [9, 10]. In addition, reductions in leptin activity and lipoprotein lipase function contribute to increased appetite and altered lipid metabolism, promoting fat accumulation [11, 12].

Deworming status (p = 0.042) and anti-ectoparasite treatment status (p = 0.024) were also significantly associated with obesity status. Cats free from endoparasite and ectoparasite infestations may experience lower energy expenditure associated with parasitic burdens, thereby favoring weight gain. Furthermore, the housing system was significantly associated with obesity status (p = 0.006). Cats maintained predominantly indoors may have reduced opportunities for physical activity, thereby increasing the risk of overweight and obesity. Previous studies have similarly reported that cats living in apartments or urban households are more likely to become obese [21]. Paul *et al*. [19] reported that 79.03% of cats routinely received deworming treatment and 83.87% were vaccinated against rabies, indicating generally good preventive healthcare practices among cat owners.

### Clinical findings and welfare implications

The overall health status of the study population was generally favorable. The prevalence of urogenital disease (3.8%), digestive disease (5.7%), respiratory disease (9.7%), and integumentary disease (11.5%) was relatively low. These findings indicate that most cats received adequate health management and veterinary care.

Integumentary disorders were primarily associated with ectoparasite infestations, which were identified in both the case group (20.2%) and the control group (32.7%). This finding may be related to housing systems that allow outdoor access, thereby increasing exposure to environmental sources of ectoparasites. Another contributing factor may be the incomplete implementation of anti-ectoparasite control measures by some owners.

Dental and periodontal disease was observed in 34.6% of cats in the case group and 32.7% of cats in the control group. The relatively high prevalence of dental disorders may indicate limited owner awareness regarding routine dental examinations and preventive oral healthcare. According to Lang [22], companion animal welfare includes appropriate veterinary attention for dental disease, urinary disorders, parasitic infections, chronic diseases, poisoning, wounds, and infectious diseases. Therefore, improving owner awareness regarding dental care may further enhance welfare outcomes in companion cats.

### Blood chemistry profiles

**Blood glucose:** Most cats exhibited blood glucose concentrations within the normal reference range. Elevated blood glucose values were observed in 3.8% of cats in the case group and 5.7% of cats in the control group. Increased feeding frequency, ad libitum feeding practices, and metabolic changes associated with sterilization may contribute to elevated glucose concentrations. Butera and Allawy [9, 10] reported that reduced estradiol concentrations following sterilization promote hyperphagia and increased food intake. Furthermore, gonadectomy may increase interleukin-6 production and activate protein kinase pathways involved in insulin resistance, thereby increasing the risk of hyperglycemia and diabetes mellitus [11, 12].

**Figure 2:**
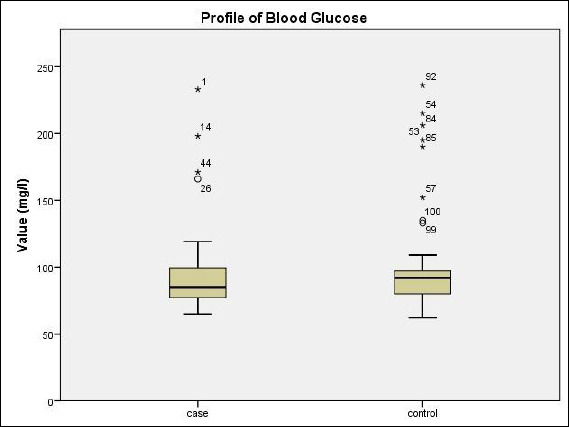
Profile of blood glucose.

Low blood glucose values observed in a small proportion of cats may be associated with hepatic dysfunction, as liver disease is a recognized cause of hypoglycemia [23]. Nevertheless, blood glucose concentrations did not differ significantly between the case and control groups (χ² = 0.990, p = 0.609), indicating that obesity status was not associated with altered glucose homeostasis in this study population (Figure 2).

**AST and ALT:** AST concentrations were within normal limits in most cats (86.5%). Elevated AST values were detected in 4.8% of cats in the case group and 7.7% of cats in the control group. AST is a hepatocellular enzyme released following liver or muscle cell injury and is commonly used as an indicator of hepatic and muscular disorders [23]. Elevated AST and ALT activities are frequently associated with hepatic necrosis and muscle damage [23]. However, no significant difference in AST concentrations was observed between groups (χ² = 1.870, p = 0.393).

**Figure 3:**
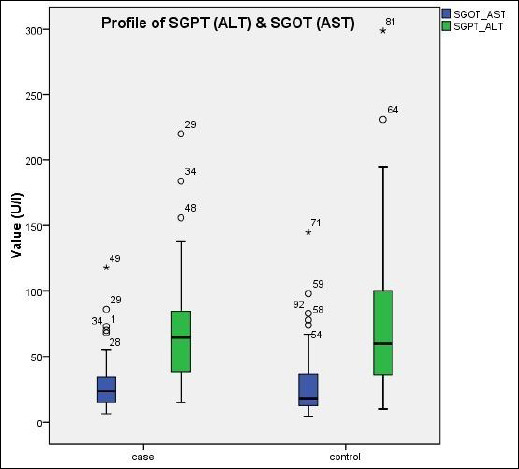
Profile of alanine aminotransferase and aspartate aminotransferase.

ALT concentrations were within normal limits in 51.8% of cats. Elevated ALT values were observed in 15.4% of cats in the case group and 20.2% of cats in the control group. ALT is considered a liver-specific enzyme in companion animals and is widely used for detecting hepatocellular injury [24]. Increased ALT activity may result from hepatocyte necrosis or alterations in cellular membrane permeability [23]. Conversely, low ALT values may occur when functional hepatic mass is insufficient to synthesize and release adequate enzyme quantities [23]. Similar to AST, ALT concentrations did not differ significantly between groups (χ² = 1.419, p = 0.492) (Figure 3).

**Figure 4:**
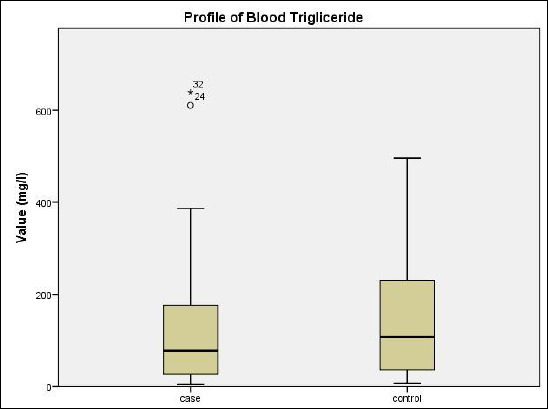
Profile of triglycerides.

**Triglyceride:** Triglyceride concentrations were within the normal range in 30.8% of cats. Elevated triglyceride concentrations were observed in 23.1% of cats in the case group and 27.9% of cats in the control group, whereas low triglyceride concentrations occurred in 9.6% and 8.6% of cats, respectively. Hypertriglyceridemia may occur in association with biliary obstruction and several metabolic disorders, including diabetes mellitus, hyperadrenocorticism, hypothyroidism, and pancreatitis, all of which may secondarily affect hepatic function [23]. Despite these observations, triglyceride concentrations were not significantly different between the case and control groups (χ² = 1.024, p = 0.599) (Figure 4).

**Figure 5:**
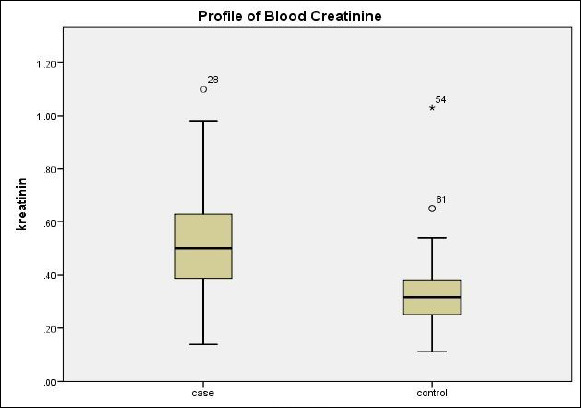
Profile of creatinine.

**Creatinine:** Creatinine concentrations were within the normal reference range in only 6.8% of cats. Low creatinine concentrations were observed in 44.2% of cats in the case group and 49.0% of cats in the control group. Creatinine is commonly included in serum biochemical panels because of its value in evaluating renal function and muscle metabolism [23]. Reduced creatinine concentrations may occur in advanced muscle wasting conditions because of reduced muscle mass available for creatinine production [23]. Nevertheless, creatinine concentrations did not differ significantly between the case and control groups (χ² = 3.829, p = 0.050) (Figure 5).

Overall, the blood chemistry profiles showed no significant differences between obese and ideal-BCS-sterilized cats. These findings suggest that many overweight-obese cats may have physiologically adapted to their increased body condition while maintaining metabolic homeostasis. Glucose homeostasis depends on coordinated regulation by pancreatic β-cells, hepatic glucose production, and peripheral glucose utilization, particularly within skeletal muscle [27]. Therefore, despite differences in BCS, the cats in the present study appeared capable of maintaining physiological equilibrium, resulting in blood chemistry profiles comparable to those of cats with ideal body condition.

### Animal welfare outcomes

Animal welfare outcomes can be evaluated according to the fulfillment of the five welfare needs: protection from pain, suffering, injury, and disease; provision of a suitable diet; provision of a suitable environment; appropriate housing with or apart from other animals; and the opportunity to express normal behavioral patterns [2].

Educational background, urban residence, age, and experience in cat ownership appeared to contribute positively to the implementation of animal welfare practices. This was reflected by high owner awareness regarding vaccination, with 95.2% of owners vaccinating all or some of their cats. Similar patterns were observed for deworming and anti-ectoparasite treatments. The low prevalence of clinical disorders affecting the urogenital, digestive, respiratory, and integumentary systems further indicates that owners generally provided appropriate preventive healthcare and disease management.

Regarding dietary welfare, all cats had unrestricted access to drinking water via either bowls or fountains. Although nutritional requirements were generally met through dry, wet, and homemade diets, feeding practices may have contributed to excessive weight gain. Frequent and *ad libitum* feeding were common in both groups and may have promoted the development of obesity. Reduced estrogen concentrations after sterilization are known to increase food intake and body weight through hyperphagia [9, 10]. Previous studies have also demonstrated that body weight and BCS increase after sterilization, even when cats receive energy intake similar to that of intact cats, emphasizing the need for dietary adjustment following sterilization [8].

Most cats also fulfilled welfare requirements related to housing and environmental conditions. Indoor and indoor-outdoor housing systems generally provided protection from adverse weather conditions while allowing access to comfortable resting areas. However, a small proportion of cats remained continuously confined in cages, which may represent a welfare concern.

Behavioral and emotional welfare outcomes were generally positive. Most owners regularly interacted with their cats through petting and play, with interactions lasting >15 min/day. These activities likely promoted positive behavioral and emotional responses, indicating that the welfare need to express normal behavior was adequately fulfilled in most cats.

## CONCLUSION

This study demonstrated that sterilized cats with overweight-obesity status (BCS 6–9) exhibited blood chemistry profiles comparable to those of sterilized cats with ideal body condition (BCS 4–5). No significant differences were observed between groups in blood glucose, AST, ALT, triglyceride, or creatinine concentrations. Although elevated triglyceride and ALT concentrations were detected in some animals, these abnormalities occurred in both groups and were not significantly associated with obesity status. These findings suggest that many overweight-obese sterilized cats may maintain metabolic homeostasis despite increased body fat accumulation.

Several management-related factors were significantly associated with obesity status, including male sex, deworming status, anti-ectoparasite treatment status, housing system, and cage management practices. Clinical examination findings indicated generally favorable health and welfare, with a low prevalence of digestive, respiratory, urogenital, and integumentary disorders. However, the high prevalence of dental and periodontal disease highlights the need for improved owner awareness regarding routine oral health care. Ectoparasite infestation was the only clinical parameter significantly associated with obesity status.

A major strength of this study was the comprehensive evaluation of animal welfare by integrating owner demographics, management practices, clinical findings, and blood chemistry parameters in sterilized cats. In addition, the case-control design enabled direct comparison between obese and ideal-BCS animals under real-world companion animal management conditions. However, several limitations should be acknowledged. The study was conducted at a single veterinary clinic, which may limit the generalizability of the findings. The cross-sectional assessment of blood chemistry parameters also precluded evaluation of long-term metabolic changes associated with obesity. Furthermore, dietary intake and physical activity were assessed primarily through owner-reported information, which may be subject to reporting bias.

Future studies should include larger multicenter populations, longitudinal monitoring of metabolic indicators, and more detailed assessments of dietary intake, energy expenditure, and endocrine biomarkers associated with obesity. Investigation of obesity-related changes in inflammatory and hormonal pathways may further improve understanding of the relationship between body condition, metabolic health, and animal welfare in companion cats.

Overall, the findings indicate that obesity in sterilized cats was more strongly associated with management-related factors than with measurable alterations in routine blood chemistry parameters. High levels of owner awareness regarding preventive health care, vaccination, parasite control, and environmental management contributed to generally satisfactory welfare outcomes. Continued emphasis on responsible feeding practices, weight management, dental care, and preventive veterinary medicine is essential to maintain the long-term health and welfare of sterilized companion cats.

## DATA AVAILABILITY

The data generated during the study are included in the manuscript.

## AUTHORS’ CONTRIBUTIONS

SDH: Conceptualization, methodology, investigation, data analysis and interpretation, writing – original draft, and writing – review and editing. WSN: Conceptualization, methodology, data analysis and interpretation, and writing – review and editing. GTM: Methodology, formal analysis, and writing – review and editing. All authors have read and approved the final manuscript.
